# Beyond Buprenorphine: Models of Follow-up Care for Opioid Use Disorder in the Emergeny Department

**DOI:** 10.5811/westjem.2020.7.46079

**Published:** 2020-11-02

**Authors:** Alister Martin, Kelley Butler, Tyler Chavez, Andrew Herring, Sarah Wakeman, Bryan D. Hayes, Ali Raja

**Affiliations:** * Massachusetts General Hospital and Harvard Medical School, Department of Emergency Medicine, Boston, Massachusetts; † University of California, Irvine, School of Medicine, Irvine, California; ‡ Harvard Medical School, Boston, Massachusetts; § Highland Hospital-Alameda Health System, Department of Emergency Medicine, Oakland, California; ¶ Massachusetts General Hospital, Department of Medicine, Boston, Massachusetts

## Abstract

Recent evidence shows that emergency physicians (EP) can help patients obtain evidence-based treatment for Opioid Use Disorder by starting medication for addiction treatment (MAT) directly in the Emergency Department (ED). Many EDs struggle to provide options for maintenance treatment once patients are discharged from the ED. Health systems around the country are in need of a care delivery structure to link ED patients with OUD to care following initiation of buprenorphine. This paper reviews the three most common approaches to form effective partnerships between EDs and primary care/addiction medicine services: the Project Alcohol and Substance Abuse Services and Referral to Treatment (ASSERT) model, Bridge model, and ED-Bridge model.

The ASSERT Model is characterized by peer educators or community workers in the ED directly referring patients suffering from OUD in the ED to local addiction treatment services. The Bridge model encourages prescribing physicians in an ED to screen patients for OUD, provide a short-term prescription for buprenorphine, and then refer the patient directly to an outpatient Bridge Clinic that is co-located in the same hospital but is a separate from the ED. This Bridge Clinic is staffed by addiction trained physicians and mid-level clinicians. The ED-Bridge model employs physicians trained in both emergency medicine and addiction medicine to serve within the ED as well as in the follow up addiction clinic.

Distinct from the Bridge Clinic model above, EPs in the ED-Bridge model are both able to screen at-risk patients in the ED, often starting treatment, and to longitudinally follow patients in a regularly scheduled addiction clinic. This paper provides examples of these three models as well as implementation and logistical details to support a health system to better address OUD in their communities.

## INTRODUCTION

There were more than 70,000 drug overdose deaths in the United States in 2017, 68% of which involved opioids, an increase of 12% from 2016.^1^,^2^ This rapid rise in opioid-related deaths has prompted swift action by the medical and public health communities to slow the epidemic and prevent further loss of life. One intervention which is known to reduce mortality from overdose and morbidity from addiction is providing medication for opioid use disorder (MOUD) with buprenorphine. Extensive research demonstrates the efficacy and effectiveness of MOUD with buprenorphine in respect to retention in treatment, reduction in illicit opioid use, decreased cravings, reduced diversion and improved social function.^3^–^6^ Additionally, data suggests that MOUD with buprenorphine after a nonfatal overdose decreases all-cause and opioid-related mortality following initiation of the drug and results in fewer future hospitalizations, ED visits and health care dollars spent among those maintained on treatment.^7^,^8^

However, while there are clear benefits for patients with opioid use disorder (OUD) engaged in treatment with MOUD, many patients find that accessing this life-saving therapy is difficult due to barriers in the addiction treatment system and current prescribing structure. These barriers include the following: an inadequate number of buprenorphine prescribers, particularly in rural areas; specialty addiction treatment clinics that don’t offer MOUD; insurance restrictions; the need for a Drug Enforcement Administration (DEA) X waiver to prescribe buprenorphine, and stigma and discrimination against MOUD. Additionally, OUD may co-occur with other psychosocial complexities that may make establishing primary care difficult. This combination of barriers and having convenient access to withdrawal management in the ED provides means that many patients use the ED as their primary source of care for opioid related issues (withdrawal symptoms, overdose, treatment seeking).^9^ There were 209 opioid related visits to the ED per 100,000 population in 2015, representing a steady rise over recent years.^9^

Recent landmark studies showed that EDs may be able to play a more active role in the management of OUD. D’Onofrio et al. showed that initiating buprenorphine during the patient’s ED visit and directly linking them with primary care follow-up doubled the percentage of patients engaged with buprenorphine treatment, as compared to those receiving only a referral and reduced the total amount of illicit opioids used in the following months.^10^ This review article outlines the various approaches to developing linkages to care for patients with OUD.

## TREATMENT WITH BUPRENORPHINE

Buprenorphine is one of three US Food and Drug Administration (FDA)-approved forms of MOUD and the only opioid agonist treatment for OUD that can be prescribed in an office-based setting. Clinical trials have demonstrated the efficacy of buprenorphine and other forms of MOUD for individuals with OUD. In a meta-analysis conducted by Mattick et al, buprenorphine was superior to placebo in retaining people in treatment in all of the 14 placebo-controlled comparisons.^2^ This finding was further supported by D’Onofrio et al through a randomized clinical trial involving 329 opioid-dependent patients who were treated at an urban teaching hospital. They found that among patients with OUD, ED-initiated buprenorphine treatment, when compared to brief intervention or referral only, significantly increased engagement in addiction treatment, reduced self-reported illicit opioid use, and decreased use of inpatient addiction treatment services.^11^ Clark et al demonstrated a 50% lower risk of relapse than behavioral treatment without MOUD.^12^ In a study of 33,923 Medicaid patients diagnosed with OUD, treatment with buprenorphine was found to be effective across a range of outcomes, including reducing all-cause mortality, improving physical and mental health, and decreasing illicit drug use. Patients treated with buprenorphine experienced a 75% reduced mortality as compared to those treated with three psychosocial interventions alone.^13^

Practically, buprenorphine is also the most realistic type of MOUD for emergency physicians (EP) to initiate. While methadone can be easily started in an ED setting continued treatment requires direct admission to an opioid treatment program, which can be challenging for EDs to coordinate particularly in areas where wait times for opioid treatment programs are long. Providers are granted the power to prescribe buprenorphine through the Drug Addiction Treatment Act of 2000. This act requires qualified clinicians to obtain a special waiver from the separate registration requirements of the Narcotic Addict Treatment Act – 1974 to treat opioid use disorder with Schedule III, IV, and V medications or combinations of such medications that have been approved by FDA for that indication.^14^ Qualified clinicians include licensed physicians, physicians assistants, nurse practitioners, clinical nurse specialists, certified registered nurse anesthetists and certified nurse-midwives. These clinicians must complete a training course (8 hours for physicians and 24 hours for all other clinicians) and submit a waiver application through the Substance Abuse and Mental Health Services Administration (SAMHSA) to the Drug Enforcement Agency to qualify for a waiver to prescribe buprenorphine.^15^ ED physicians to obtain waviers yet, only 1% of all emergency physicians have this waiver.^16^, ^17^

## FOLLOW-UP

ED initiation of buprenorphine is optimized through connection to effective, outpatient follow up options. Importantly, as EDs look to expand the services they provide for OUD, patients lack of timely follow-up for continued prescribing could pose a significant, yet surmountable, barrier. Thus far there have been several models that have been used throughout the country. We review three of these models below in detail and summarized in Table 1.

### Model 1: ASSERT Model

The Project Alcohol and Substance Abuse Services Education and Referral to Treatment (ASSERT) model is characterized by peer educators or community workers in the ED directly referring patients found to be suffering from OUD in the ED to local addiction treatment services.

#### Background

The ASSERT model was first developed, implemented and tested at the Boston Medical Center (BMC) in 1995. Health Promotion Advocates (HPAs) are at the center of this model. HPAs are alcohol and drug treatment counselors certified by the Massachusetts Department of Public Health. They are linguistically and ethnically suited to meet the needs of the Boston Medical Center (BMC) patient base, well-versed in interview and screening tools and most importantly, members of the communities the project aimed to serve.

#### Examples of Implementation

On service daily in the ED from 9AM to 11PM, the HPAs are charged with screening patients suspected to be suffering from substance use disorder and enrolling them in Project ASSERT. Following initial screening and retrieval of informed consent, the HPAs then engage in a trauma-informed, non-judgmental conversation utilizing the Brief Negotiated Interview algorithm developed at BMC. This interaction is primarily a motivational interview that focuses on patients’ cultural background, beliefs, values, and readiness to engage in treatment. Given the extra time afforded by their role, HPAs get to know the patients on a deeper level often unachievable by EPs and thus provide a service not previously available. Together with the patient, the HPAs craft a harm reduction plan. The HPAs may refer patients to local resources such as in-patient detoxification programs, methadone clinics or outpatient centers in the surrounding area where they can begin MOUD. These advocate teams do not directly supply the medication but rather serve as a knowledgeable source of information about the resources patients can utilize immediately after their ED visit. The patients also agree at enrollment to follow up with the HPA after 60 days.

A 1997 study by Bernstein et al. found that 18% of the total ED population for the study period was screened and a substance use problem was detected in 41% of those patients.^18^ OF those detected patients, 37% enrolled in Project ASSERT. For those enrolled, an average of 1.2 referrals were made per patient over the course of the study period, many for basic health screenings like mammograms or referrals to primary care clinics. The study found that many of the patients screened lacked a regular primary care physician and utilized the ED to seek care. At the 60- to 90-day follow-up visit, patients reported keeping over half of appointments made to the Boston Office of Treatment Improvement Central Intake, inpatient facilities and outpatient services, Narcotics Anonymous, or Alcoholics Anonymous. Patients also reported a reduction in quantity and/or frequency of drug use for the 2 months preceding the follow-up visit, compared with the 2 months before enrollment. Some stopped using altogether.

The Boston Medical Center has continued to see success throughout the program’s duration, enrolling tens of thousands of patients since its implementation. In 2016, for example, BMC successfully placed 56% of patients who were requesting acute treatment for substance use disorder through detox.^19^ Today, their Faster Paths to Treatment program utilizes the ASSERT model of directly evaluating, motivating, and referring patients with substance use disorder to a comprehensive care network of inpatient and outpatient detoxification, treatment, and aftercare services integrated with mental health and medical care.

The Yale New Haven Hospital was similarly successful in implementing an ASSERT model of peer educator-based referrals. Patients directly referred by Project ASSERT were found to be twice as likely to enroll in a specialized treatment center. Additionally, 55% of patients referred to a specialized treatment center through Project ASSERT successfully enrolled within one month of referral.^20^ As of 2018, Project ASSERT at Yale had screened over 50,000 patients since its implementation in 1999 and is now distributing life-saving naloxone to hundreds of families in the community.^21^

#### Benefits, Logistics, and Limitations of this Model

Each of these permutations of the ASSERT model necessitates dedicated community health workers or peer support staff versed in addiction, motivational interviewing and trauma-informed care to be physically present in the ED. Licensure and scope of practice for these support staff vary considerably between states which may explain the variability seen within successful models. Health systems considering implementation of the ASSERT model must consider the hours that these staff will be present, hiring practices and support structures for peer support staff, how these support staff communicate with clinicians and what local resources are at the ED’s disposal for patients with OUD. Peer support staff support patients in their efforts to seek relief in the ED and support clinicians in their attempts to meet the complex psychosocial needs of addicts in settings where they are often ill-equipped in terms of training, comfort and capacity. This model may not be effective if community resources are not robust enough to support longitudinal care for patients with OUD.

### Model 2: Bridge Model

The second model of care examined is the Bridge model. In this model, prescribing physicians in an ED screen patients for OUD, provide a short term prescription for buprenorphine, and then refer the patient directly to an outpatient clinic called a Bridge Clinic that provides MOUD. The Bridge Clinic is co-located in the same hospital but is a separate from the ED and is staffed by addiction trained physicians and mid-level clinicians.

#### Background

In contrast to the ASSERT model, which largely relies on peer support staff in identifying and facilitating the referral of patients from the ED to addiction services, the Bridge model relies on the diagnostic and prescribing capacity of the EP, sometimes with the support of peers. This prescribing power is granted by obtaining a DEA X Waiver.^22^ This necessitates completing an 8-hour course approved by the Substance Abuse and Mental Health Services Administration. Allied health professionals must meet a 24 hour training requirement. The Yale New Haven Hospital and Massachusetts General Hospital are the first hospital EDs to have the majority of their physicians waivered to prescribe buprenorphine following an in-house training.^23^ Once X-waivered, physicians engaged in the Bridge Model of treatment are responsible for identifying patients with OUD.

#### Examples of Implementation

The most notable example of the Bridge Clinic Model is the Massachusetts General Hospital OUD program, which became the first hospital in the state to offer seamless ED-initiated buprenorphine with rapid next day follow up in its Bridge Clinic. EPs in this program engage with the patient, assessing their interest in buprenorphine treatment, and offering initiation while in the ED. The physician then facilitates a “warm” hand-off to the Bridge Clinic, an outpatient site in the same hospital system well versed in the longitudinal treatment and management of OUD. This clinic is available to see patients within normal business hours including weekends.

During Bridge Clinic hours, patients are discharged directly from the ED to the Bridge Clinic where they can begin or continue buprenorphine and continue accessing addiction services. Those discharged in the evening or overnight are given a two day supply of buprenorphine called a home pack, or a prescription for buprenorphine, to be taken at home and are instructed to return to the Bridge Clinic the next day for ongoing treatment.

Mid Coast Hospital in Brunswick, ME provides another example of the Bridge Clinic model. Its program became the first hospital in Maine to prescribe buprenorphine in its ED leveraging a Bridge Clinic for follow up. Patients suffering from OUD who are seen in the ED at this hospital are evaluated by ED physicians and are referred to Mid Coast Hospital’s Addiction Resource Center (ARC) program. In the majority of cases patients leave the ED with an appointment at the ARC on the next business day.

#### Benefits, Logistics, and Limitations of this Model

To be maximally effective the Bridge model requires a substantial proportion of ED clinicians within the department to be X-waivered. The training requirement, while minimal, remains a barrier for clinicians to prescribing MOUD with buprenorphine. This barrier can be overcome on a case by case basis as the DEA allows a 72-hour exemption that permits non-waivered prescribers to administer buprenorphine or methadone while arranging linkage to ongoing treatment. Thus, variations of the Bridge model are feasible even when ED physicians do not have their waiver. These clinicians may instead use the 72-hour exemption to administer one dose of buprenorphine prior to discharge of patients in withdrawal and arrange direct follow-up in a Bridge Clinic. The clinician must, however; be comfortable engaging with patients that present with needs specific to OUD. They must be versed in the referral process of the health system’s Bridge Clinic and be able to engage in motivational interviewing to support the patient in addressing their health needs.

The Bridge model is most defined by the ability for providers, waivered or not, to immediately connect a patient to a clinic that is co-located to the ED. This co-location supports low-barrier access to evidence-based treatment because the clinic provider has access to notes written by the ED clinician(s), can connect with the patient when they aren’t in acute withdrawal and can help a patient along their journey to recovery that was already jumpstarted by the EP. This Bridge Clinic requires addiction-trained clinicians capable of prescribing MOUD, peer support staff to address the patients’ accompanying psychosocial needs, connections to therapists and physical space within the hospital or health system that is accessible immediately from the ED.

The Bridge Model seeks to treat OUD like any other acute medical condition treated in the ED by providing low-threshold follow-up care by a separate, highly trained specialist in the outpatient setting within the same hospital as the referring ED.^24^

### Model 3: ED-Bridge

The final model we will examine is the ED-Bridge model, a novel system that employs physicians trained in both Emergency Medicine and Addiction Medicine to serve within the ED as well as in the follow up addiction clinic.

#### Background

As opposed to the Bridge Clinic model detailed above, EPs in this model are both able to screen at-risk patients in the ED, often starting treatment in the ED, and are also able to longitudinally follow patients in a regularly scheduled addiction clinic. The unique feature of this model relies on the fact that the majority of clinicians that engage in it are EPs who also board certified in addiction medicine. This added expertise allows for continuity of care and a consistent patient-provider relationship.

#### Examples of Implementation

One notable application of this approach is a clinic run by Dr. Andrew Herring in Oakland, CA at Highland Hospital. Physicians within the ED at Highland Hospital are trained to identify and screen patients for OUD. Key addiction specialty-trained EPs among this group act as both gateways to addiction treatment and longitudinal care clinicians by first offering patients buprenorphine therapy in the ED, if applicable, and access to the follow up clinic appointments during regularly scheduled weekly clinic times.^25^ In this model, EPs like Dr. Herring are able to utilize these clinic times to follow patients through the first parts of their recovery journey after engaging with them in the ED.^26^ This clinic is staffed by EPs with addiction training as well as by substance use navigators who are staff tasked with providing motivation and reassurance, and who address all manner of issues ranging from transportation to childcare and dealing with landlords and legal issues.^27^

Another example of this longitudinal model led by EPs who are able to fill both roles is the Upstate Emergency Opioid Bridge Clinic program which is led by Dr. Ross Sullivan, an EP who is also board certified in addiction medicine at the State University of New York Upstate Medical University in Syracuse. This clinic operates twice a week from the Downtown Campus and is housed in a space adjacent to the ED. Patients who are started on buprenorphine in the ED are then referred to the clinic for a follow-up visit within one to three days. Along with EPs who continue to prescribe buprenorphine for patients longitudinally, this clinic is staffed by peer specialists who provide information and encouragement as well as helping to address the broader social determinants of health such as finding housing and accessing Social Security benefits.^28^

#### Benefits, Logistics, and Limitations of this Model

All the aforementioned benefits and considerations for the Bridge Clinic model remain with a significant addition in the ED-Bridge model: the ED-Bridge physicians also see patients in clinic thereby leveraging the therapeutic relationship created by the emergency physician during the initial ED encounter. Given this, the ED-Bridge model moves beyond requiring that EPs feel comfortable having conversations with patients about addiction. Instead, it calls for the EPs to be trained in providing treatment for addiction and necessitates that they be X-waivered at a minimum. Very few EPs are formally trained—let alone board certified in addiction medicine—in this manner which may present a significant barrier for hospitals and health systems. Outpatient clinic settings can be foreign for classically trained EPs which supports the need for additional addiction training.

The ED-Bridge Model, exemplified by these two programs, seeks to blend the roles of emergency and addiction physicians offering an opportunity to provide longitudinal care and a basic way to continue to prescribe patients buprenorphine in the immediate period following ED evaluation. The CA Bridge program offers focused technical assistance and training for any hospital or health care facility in the United States to design their own ED-Bridge model.^29^

## SUMMARY RECOMMENDATIONS

The unique contextual features of the hospital system and ED where these follow-up models could be implemented will necessarily impact the decision to choose one over the others. Given the peer-centered approach, which leverages less highly skilled advocates rather than clinicians, the ASSERT model is a viable solution for departments which do not have the resources to support ED clinician waiver training or hospital systems that are not interested in investing in a functional Bridge Clinic. The drawbacks of this model, however, are that ED clinicians are not the central point of contact for patients with regard to their use disorder while they are in the ED. This results in ED providers potentially building a reliance on the advocates to interface with patients with opioid use disorder rather than developing the vocabulary and skillset to address these issues themselves.

The Bridge model offers a convenient patient experience as the addiction clinic is often co-located on the same floor as the ED or within walking distance, allowing for logistically easier handoffs to addiction treatment teams post-ED discharge. This co-location potentially reduces the likelihood of no-shows and reduces the barriers to entry into evidence-based treatment clinics. The cost of initiating a Bridge Clinic include but are note limited to; the physical clinical space, clinician time, and administrative support. These costs are nontrivial. For hospitals with limited resources the Bridge Clinic model could represent a significant time and cost investment over and above what is feasible.

The ED-Bridge model offers a near-seamless patient experience of patient-provider engagement given the consistency of who the prescribing clinician is in the ED and the longitudinal prescriber in the addiction clinic. The significant downside in this model, however, is the hyper-specialization required to make this model viable. The ED clinician must not only be a board-certified EP but must also have, in the majority of cases, board certification in addiction medicine. This emphasis on highly specialized clinicians may preclude this from being a realistic model in anything other than academic or large, urban medical centers.

## CONCLUSION

Opioid use disorder is a treatable condition and yet our healthcare system currently lacks access to provide patients with seamless ways of accessing treatment. Treatment with MOUD reduces mortality and improves the likelihood of disease remission; yet most patients with OUD never receive these lifesaving medications. As outlined above, EDs play a critical role in the effective screening, treatment initiation, and direct linkage to care for patients with OUD. A recent body of evidence shows emergency physicians can expand both their role and effectiveness in creating this link by providing buprenorphine directly in the ED. With this new knowledge, EDs around the US are in need of a framework for better treating and referring patients who present to the ED with OUD.

The current structures of the relationship between EDs and addiction services are variable around the country. This review article has outlined some of the most impactful approaches currently in place including the ASSERT model, Bridge model, and the ED-Bridge model. These models are constantly being improved but can serve as a template for the development of emergency-addiction linkages in other communities. Future research should aim to assess the effectiveness of each of these designs as well as to understand which models work best in specific populations or settings. Further work should seek to inform and equip EDs around the nation with a guide for setting up life-saving linkages between EDs and sustainable outpatient addiction care with MOUD in their respective communities.

## Figures and Tables

**Table 1:** Comparison of three models that link emergency care and addiction treatment.

Model	Description	Benefits	Challenges
ASSERT model	Peer support staff or community health workers in the ED directly refer patients with OUD to local addiction treatment services.	Peer-centered approach Leverages community resources rather than creating resources in the hospital system	Limited by community resources ED clinicians are not the primary staff members interacting with the patient on their use disorder, thereby potentially displacing the responsibility of treating patients with OUD in the ED onto other providers Licensure and scope of practice for the support staff vary considerably between states
Bridge model	Prescribing physicians in the ED screen patients for OUD, provide a short-term prescription for buprenorphine, and then refer the patient directly to an outpatient Bridge clinic that is co-located in the same hospital but is separate from the ED.	Co-location of ED and Clinic potentially reduces likelihood of no-shows Reduced barriers to entry into evidence-based clinic Communication through shared EHR	Clinic capacity is a constraint Excellent coordination between ED and Clinic is paramount to establish effective handoff Significant investment required by health system to create the Bridge Clinic Cost of the 8-hour waiver training for ED clinicians No continuity of care between prescribing clinician in the ED and prescribing clinician in the Bridge Clinic
ED-Bridge model	Physicians trained in both emergency medicine and addiction medicine both screen at-risk patients in the ED, often starting treatment in the ED, and also are able to longitudinally follow patients in the outpatient setting.	Enhanced continuity of care Decreased need for a separate, trained workforce of outpatient addiction clinicians	Highly specialized emergency physicians double boarded in emergency medicine and addiction medicine, leading to a limited supply of providers Likely limited to major urban/academic centers

*ED*, emergency department; *OUD*, opioid use disorder; *EHR*, electronic health records.
